# Supplementary material for: Genetic interactions between the chromosome axis-associated protein Hop1 and homologous recombination determinants in Schizosaccharomyces pombe
Source: Curr Genet. 2018 Mar 17;64(5):1089–104. doi: 10.1007/s00294-018-0827-7 (PMC6153652; doi:10.1007/s00294-018-0827-7)
Supplement: Supplementary file 1 — Supplementary material 1 (DOCX 756 KB) [file 294_2018_827_MOESM1_ESM.docx]

**SUPPLEMENTARY MATERIALS**

**Genetic interactions between the chromosome axis-associated protein Hop1 and homologous recombination determinants in *Schizosaccharomyces pombe***

Simon David Brown^1,2^, Olga Dorota Jarosinska^1^, and Alexander Lorenz^1^

^1^Institute of Medical Sciences (IMS), University of Aberdeen, Aberdeen AB25 2ZD, United Kingdom

^2^Present address: MRC Institute of Genetics & Molecular Medicine, University of Edinburgh, Edinburgh EH4 2XU, United Kingdom

**Specifics of yeast strain and plasmid construction**

Construction of the *kanMX6*-marked *hop1*Δ-*1* allele has been described previously (Latypov et al. 2010), the *natMX6*-marked *hop1*Δ-*25* was derived from this using an established marker swap protocol (Sato et al. 2005; Lorenz 2015).

To produce *hop1*-alleles expressing a particular splice variant and the *hop1*-Δ*HORMAD* mutant, we first partially deleted *hop1* retaining the coding sequence directly upstream of the first and directly downstream of the last intron using a *ura4* marker (Grimm et al. 1988). This targeting cassette was created by NEBuilder assembly of a PCR product representing 437bps upstream of the first intron in *hop1* (oligonucleotides oUA213 5’-GAACGCGGCCGCCAGCTGaagcttCAAATAGCGTCCATTCTACCC-3’ and oUA214 5’-GCTGCtctagaCGCTTATAGCATAATGTTGATATCGC-3’ on ALP714 genomic DNA), and a PCR product representing 314bp downstream of the sixth intron (oligonucleotides oUA215 5’-GCTATAAGCGtctagaGCAGCTATGCTGATGCCAAAAG-3’ and oUA216 5’- GCGCCTTAATTAACCCGGGGATCCTTAAATCC-3’ on a plasmid carrying the coding sequence of *hop1*), into a pFA6a vector backbone linearized by a *Bam*HI-*Hin*dIII digest. The resulting plasmid (pALo190) was linearized by an *Xba*I digest, and a *ura4* marker (PCR using oligonucleotides oUA224 5’-ACATTATGCTATAAGCGTCTAGAagctacaaatcccactggc-3’ and oUA225 5’-GGCATCAGCATAGCTGCtctagAgtgatattgacgaaactttttg-3’ on a plasmid carrying a functional *ura4* open reading frame) was then inserted by NEBuilder assembly to give pALo192. The transformation cassette was released from pALo192 by a *Bam*HI-*Hin*dIII digest and transformed into the standard lab strain FO652. This resulted in strain UoA725 carrying the *hop1-3* allele which was subsequently transformed with the cDNA of one of the two splice variants (amplified by PCR from appropriate plasmids using oligonucleotides oUA258 5’-ATGAATTCATACAAAGAGGAAATTC-3’ and oUA259 5’-TTAAATCCTCAGAGGTTTCATG-3’). The *hop1-*Δ*HORMAD* construct was made by NEBuilder assembly through merging a fragment encompassing the first 30bps of *hop1* coding sequence plus 334bps of upstream flanking sequence (amplified by PCR using oligo- nucleotides oUA385 5’-ccgtcgacctgcagccaagcttGTCCATTCTACCCAAAAGCG-3’ and oUA386 5’-CCATTGTTTGATTCATATTGCAGAATTTCCTCTTTGTATGAATTCAT-3’ on ALP1594 genomic DNA), and a fragment representing coding sequence downstream of the HORMA domain (PCR-amplified using oligonucleotides oUA387 5’-TATGAATCAAACAATGGTATTGCG-3’ & oUA388 5’-cgttgtaaaacgacggccagtgccaagcttTTAAATCCTCAGAGGTTTCATGG-3’ from a plasmid carrying the *hop1-sv1* cDNA), with *Hin*dIII-linearized pUC8. The *hop1-*Δ*HORMAD* construct was released from the resulting plasmid (pSDB8) by a *Hin*dIII digest, and transformed into UoA725. Correct integration of the splice variants and the *hop1-*Δ*HORMAD* construct was monitored by selection for FOA-resistance and verified by colony PCR and DNA sequencing (Source BioScience plc, Nottingham, UK). All strains carrying the *hop1-sv1*, the *hop1-sv2*, or *hop1-*Δ*HORMAD* alleles are derivatives of the original parental strains UoA746, UoA747, or UoA938, respectively.

Epitope-tagging of Hop1 with 13myc was achieved by constructing an appropriate transformation cassette: Using NEBuilder assembly PCR products representing the 3’ end (oligonucleotides oUA266 5’-GAAGCTTCGTACGCTGCAGGGCAGCTATGCTGATGCCAAAAG-3’ and oUA267 5’-AATTAACCCGGGGATCCGTCgagcAATCCTCAGAGGTTTCATGGTG-3’ on a plasmid carrying the coding sequence of *hop1*) and downstream flanking sequence (oligonucleotides oUA268 5’-CTCGAATTCATCGATGATCTTATACGACTTATCCTATCTTC-3’ and oUA269 5’-GCCACTAGTGGATCTGATCAAGACTCTATTTAACTGGG-3’ on genomic DNA of ALP1596) of the *hop1* open reading frame were inserted into pFA6a-*13myc*-*kanMX6* (Bähler et al. 1998) digested with *Sal*I and *Eco*RV. The resulting plasmid, pALo205, was verified by DNA sequencing (Source BioScience plc). A transformation cassette was amplified from pALo205 by PCR (using oligonucleotides oUA271 5’-GCAGCTATGCTGATGCC-3’ and oUA272 5’-GGATAGAACACTCTCACTTCC-3’) and transformed into strains UoA746 and UoA868. Correct integration was monitored by selection for G418-resistance and verified by colony PCR and DNA sequencing (Source BioScience plc); all strains carrying the *hop1-sv1*::*13myc-kanMX6*, the *hop1-sv2*::*13myc-*kanMX6, or the *hop1-*Δ*HORMAD*::*13myc-*kanMX6 allele are derived by crossing from the original transformants (UoA782, UoA927, UoA941).

A deletion cassette for *aho1* was constructed by cloning an upstream flanking sequence of *SPAC688.03c* (PCR using oligonucleotides oUA13 5’-ttaattcagctgACAAGGCTTCAGCAAGC-3’ and oUA14 5’-ttaattggatccGTAGTAGCAATTGCCTACG-3’ on genomic DNA of ALP1594) digested with *Pvu*II and *Bam*HI into pAG25 (Goldstein and McCusker 1999) linearized with *Pvu*II and *Bam*HI. The resulting plasmid (pALo115) was linearized by digesting with *Sac*I and *Spe*I and a downstream flanking sequence of *SPAC688.03c* (PCR using oligonucleotides oUA15 5’-ttaattgagctcGCGTAATTAAGCTATCGC-3’ and oUA16 5’-ttaattactagTACTGTATACTCACCTCG-3’ on genomic DNA of ALP1594) digested with *Sac*I and *Spe*I was inserted by standard cloning to give pALo116. The transformation cassette was released by a *Pvu*II-*Spe*I digest and transformed into the standard lab strain FO652. This construct removes the entire *aho1* coding sequence plus 11bps upstream and 41bps downstream. Correct integration was monitored by selection for CLONNAT-resistance and verified by PCR; all strains carrying the *aho1*Δ-*84*::*natMX4* deletion are derived by crossing from the original transformant (UoA423).

To over-express Aho1, the coding sequence of *SPAC688.03c* was amplified by PCR (using oligonucleotides oUA99 and oUA100 on genomic DNA of UoA473) digested with *Nde*I and *Bam*HI and cloned into pREP41-*eGFPC* (Craven et al. 1998) linearized by an *Nde*I-*BamH*I digest. The construct was verified by DNA sequencing (Source BioScience plc).

To make plasmids for over-expression of Hop1 in *Sz. pombe* we cloned a PCR product representing genomic or cDNA versions of the *hop1* coding sequence (using oligonucleotides oUA86 5’-aattaaCTCGAGATGAATTCATACAAAGAGGAAATTC-3’ and oUA87 5’-attaaggatccTTAAATCCTCAGAGGTTTCATG-3’ on genomic DNA of ALP1594 and on meiotic cDNA of UoA399) after an *Xho*I-*Bam*HI digest into pJR-41XL (Moreno et al. 2000) linearized with the same restriction endonucleases. Inserts were verified by DNA sequencing (Source BioScience plc), and we found discrepancies with the coding sequence prediction on PomBase (<https://www.pombase.org/>) (see Results section for details). To corroborate our initial finding we also NEBuilder-assembled meiotic cDNA clones from UoA399 and UoA722 amplified by PCR (using oligo- nucleotides oUA279 5’-GCTATGACCATGATTACGATGAATTCATACAAAGAGGAAATTC-3’ and oUA280 5’-CTTGGCTGCAGGTCGACGTTAAATCCTCAGAGGTTTCATG-3’) into pUC8 linearized by a *Bam*HI-*Eco*RI digest. The relevant 5’ portion of *hop1* was sequenced in a total of 25 clones.

All DNA modifying enzymes (high-fidelity DNA polymerase Q5, restriction endonucleases, T4 DNA ligase) and NEBuilder HiFi DNA Assembly Master Mix were obtained from New England BioLabs. Oligonucleotides were supplied by Sigma-Aldrich Co. (St. Louis, MO, USA).

All plasmid sequences are available online as supporting material (<https://doi.org/10.6084/m9.figshare.5373163.v2>, Lorenz 2018).

**a**

**ATGAATTCATACAAAGAGGAAATTCTGCAAACTAAGTCTGACTTTACTCTTAAAAATTTAATTTTCTTCGCGATATCAACATTATGCTATAAGCG**GTACGCAGACATATAATTTTTAAATTAAAATAGCTAACAGAAGGCAAG**TGCTCTTTTCAATGAAAACTGCTACAAGAAAGTCAATTTCGAAATTGAACATTTTAAAGGAGCTGACTTTGATTGTCAATTGAAACCTACTGTGGTTTCTTTACAAGCTGGAGTGGACAAAGAAGCAGACAGTTTTTTGGAAATGATG**GTGAGTGTTTAAAACTTATCTAAAGAAATAAACTAATATTTTAG**AAAACTTATATTTTCTCACTTGTATCAATGAAAGTTCCATTTACTGTTTACCTGATTATTTCGTCACAATGCAAGTCAATATTAGAGGATGACGCGGTTGAAAAGGAGATATTTTCATTTACAATTAACCCTGGAAGTGAAGAGAAAATATGTTGTGAGTCTTTTGTTT**GTAAATTTTTTAACAGACTTCATGAGAGTTTTATCAAG**CTTATCAACGTTCAGAAAGGTTTGTAATAAAGTTATTTTTAAGTGGTAACGTAAAAACAGAGTGCAAAGACGAAGAAAAAGTCGTACAAATTATTACAAAAATGGAACGCTTTCAATTATCAAAAGGAGAAGCAACTAAAGCTGGCGTGTTTTTAAATACCGTAGAAACAAAAGATTGCATGTCTTGGCTCAATCGAGGAGAATTCAAAGATATTGTATCTTTTTATGAATCAAACAATGG**GTAAGTAAATTAAAACAAATTTCAAGTTACTAATCTATAG**TATTGCGATTTCACATTGTTCTCATGCATTTGTACCGATAAGTACAGAGAAGATCATGATTAATAAAGAATCATCATTATTTGATTCTCAAGAAAAAATTGATTCTCAGCTCGAAAAGTTCCTCCAGCCTTTGAAATACGATGAAATAGGCTCAACTCAAATATTGGATGAACAATCAGTTGAAAAAAGTTTGAGTCAAGGAAAATGTGAGAAAATGCAGAATGAAAGTCGTGGATTAAGGGAAATAAAAAATAACAATCCTTGTGAAGAAGTAAAGAAAAGCAATTGGTTAAAAAAAAACATTTCAGGGAGTGACAAAGTTGACAAAGCCGAAAAGAAAAAAGCACTTTTGAATTGTGAATGCGGTGATTCGACGGAAGACTCAGAAATG**GTGTGTAATCGTTTATACCTGCTAAAGACTAACAATATTTTACACAG**TTTCAATGCGAAAGATGTGATGGATGGGTTCACTGTGCTTGCTATGGATTTGAGAGTGACTCTGATCCTCGCCAACCAAACCAATTGTTATGTTATACATGTTTGTTAGTTGATTCTGAATCTAGTTTGTATGACCGAATGACCATGTTGGTCGCATACAGACGAGCAATTCGGTGTATTTGGGCGTCAGAGTATCAAGGGTTCCAAAAGCTAGCGGCGCGACTGA**GTAAGGAAATTTACCAACTCATTGACCATATACTAACATTATCTAAG**ATTGCAGCTATGCTGATGCCAAAAGAATTGAAGAAAGATTAGTTAACGAAAACATAATCTATAAGGAAAAAAAACGAAAATGGATATATTTTACGAATAAAAGCCCTGAAATGGTGTCATATCTTCGTGAGAAGTATTTTACACCGTCGCGATGGATATCTCATCTGAATTTTCAAAATTATCGTCAAGAGAATCAACGTGTAAATATGCGATCTTTTCTACGGCCCGAAAGAATGGAAGTAATAGAAAGACCAAAAAAGGTTTCTAAGACTTCAAACACTAAAGAGACAGACACCATGAAACCTCTGAGGATTTAA**

**b**

**ATGAATTCATACAAAGAGGAAATTCTGCAAACTAAGTCTGACTTTACTCTTAAAAATTTAATTTTCTTCGCGATATCAACATTATGCTATAAGCG**GTACGCAGACATATAATTTTTAAATTAAAATAGCTAACAGAAGGCAAG**TGCTCTTTTCAATGAAAACTGCTACAAGAAAGTCAATTTCGAAATTGAACATTTTAAAGGAGCTGACTTTGATTGTCAATTGAAACCTACTGTGGTTTCTTTACAAGCTGGAGTGGACAAAGAAGCAGACAGTTTTTTGGAAATGATG**GTGAGTGTTTAAAACTTATCTAAAGAAATAAACTAATATTTTAG**AAAACTTATATTTTCTCACTTGTATCAATGAAAGTTCCATTTACTGTTTACCTGATTATTTCGTCACAATGCAAGTCAATATTAGAGGATGACGCGGTTGAAAAGGAGATATTTTCATTTACAATTAACCCTGGAAGTGAAGAGAAAATATGTTGTGAGTCTTTTGTTTGTAAATTTTTTAACAGACTTCATGAGAGTTTTATCAAGCTTATCAACGTTCAGAAAG**GTTTGTAATAAAGTTATTTTTAAGTGGTAACGTAAAAACAG**AGTGCAAAGACGAAGAAAAAGTCGTACAAATTATTACAAAAATGGAACGCTTTCAATTATCAAAAGGAGAAGCAACTAAAGCTGGCGTGTTTTTAAATACCGTAGAAACAAAAGATTGCATGTCTTGGCTCAATCGAGGAGAATTCAAAGATATTGTATCTTTTTATGAATCAAACAATGG**GTAAGTAAATTAAAACAAATTTCAAGTTACTAATCTATAG**TATTGCGATTTCACATTGTTCTCATGCATTTGTACCGATAAGTACAGAGAAGATCATGATTAATAAAGAATCATCATTATTTGATTCTCAAGAAAAAATTGATTCTCAGCTCGAAAAGTTCCTCCAGCCTTTGAAATACGATGAAATAGGCTCAACTCAAATATTGGATGAACAATCAGTTGAAAAAAGTTTGAGTCAAGGAAAATGTGAGAAAATGCAGAATGAAAGTCGTGGATTAAGGGAAATAAAAAATAACAATCCTTGTGAAGAAGTAAAGAAAAGCAATTGGTTAAAAAAAAACATTTCAGGGAGTGACAAAGTTGACAAAGCCGAAAAGAAAAAAGCACTTTTGAATTGTGAATGCGGTGATTCGACGGAAGACTCAGAAATG**GTGTGTAATCGTTTATACCTGCTAAAGACTAACAATATTTTACACAG**TTTCAATGCGAAAGATGTGATGGATGGGTTCACTGTGCTTGCTATGGATTTGAGAGTGACTCTGATCCTCGCCAACCAAACCAATTGTTATGTTATACATGTTTGTTAGTTGATTCTGAATCTAGTTTGTATGACCGAATGACCATGTTGGTCGCATACAGACGAGCAATTCGGTGTATTTGGGCGTCAGAGTATCAAGGGTTCCAAAAGCTAGCGGCGCGACTGA**GTAAGGAAATTTACCAACTCATTGACCATATACTAACATTATCTAAG**ATTGCAGCTATGCTGATGCCAAAAGAATTGAAGAAAGATTAGTTAACGAAAACATAATCTATAAGGAAAAAAAACGAAAATGGATATATTTTACGAATAAAAGCCCTGAAATGGTGTCATATCTTCGTGAGAAGTATTTTACACCGTCGCGATGGATATCTCATCTGAATTTTCAAAATTATCGTCAAGAGAATCAACGTGTAAATATGCGATCTTTTCTACGGCCCGAAAGAATGGAAGTAATAGAAAGACCAAAAAAGGTTTCTAAGACTTCAAACACTAAAGAGACAGACACCATGAAACCTCTGAGGATTTAA**

**Figure S1. *hop1* coding sequence** (**a**) as predicted and annotated on PomBase (<https://www.pombase.org/>, last accessed on 03/06/2017), (**b**) as determined experimentally (*hop1-sv1*); exons in bold, introns in regular font. The area of discrepancy between the annotation and the actual cDNA sequence in the 3^rd^ intron is shown in red/pink, and the 21 nucleotides potentially removed by an alternative splicing event using a downstream AG as acceptor site in *hop1-sv2* are highlighted in grey in the *hop1-sv1* sequence (b).

**a**

**MNSYKEEILQTKSDFTLKNLIFFAISTLCYKRALFNENCYKKVNFEIEHFKGADFDCQLKPTVVSLQAGVDKEADSFLEMMKTYIFSLVSMKVPFTVYLIISSQCKSILEDDAVEKEIFSFTINPGSEEKICCESFVSYQRSERFVIKLFLSGNVKTECKDEEKVVQIITKMERFQLSKGEATKAGVFLNTVETKDCMSWLNRGEFKDIVSFYESNNGIAISHCSHAFVPISTEKIMINKESSLFDSQEKIDSQLEKFLQPLKYDEIGSTQILDEQSVEKSLSQGKCEKMQNESRGLREIKNNNPCEEVKKSNWLKKNISGSDKVDKAEKKKALLNCECGDSTEDSEMFQCERCDGWVHCACYGFESDSDPRQPNQLLCYTCLLVDSESSLYDRMTMLVAYRRAIRCIWASEYQGFQKLAARLNCSYADAKRIEERLVNENIIYKEKKRKWIYFTNKSPEMVSYLREKYFTPSRWISHLNFQNYRQENQRVNMRSFLRPERMEVIERPKKVSKTSNTKETDTMKPLRI***

**b**

**MNSYKEEILQTKSDFTLKNLIFFAISTLCYKRALFNENCYKKVNFEIEHFKGADFDCQLKPTVVSLQAGVDKEADSFLEMMKTYIFSLVSMKVPFTVYLIISSQCKSILEDDAVEKEIFSFTINPGSEEKICCESFVCKFFNRLHESFIKLINVQKECKDEEKVVQIITKMERFQLSKGEATKAGVFLNTVETKDCMSWLNRGEFKDIVSFYESNNGIAISHCSHAFVPISTEKIMINKESSLFDSQEKIDSQLEKFLQPLKYDEIGSTQILDEQSVEKSLSQGKCEKMQNESRGLREIKNNNPCEEVKKSNWLKKNISGSDKVDKAEKKKALLNCECGDSTEDSEMFQCERCDGWVHCACYGFESDSDPRQPNQLLCYTCLLVDSESSLYDRMTMLVAYRRAIRCIWASEYQGFQKLAARLNCSYADAKRIEERLVNENIIYKEKKRKWIYFTNKSPEMVSYLREKYFTPSRWISHLNFQNYRQENQRVNMRSFLRPERMEVIERPKKVSKTSNTKETDTMKPLRI***

**Figure S2. Hop1 translated coding sequence** (**a**) as predicted and annotated on PomBase (<https://www.pombase.org/>, last accessed on 03/06/2017), and (**b**) as derived from experimental cDNA sequencing data for the long splice variant Hop1-sv1. The discrepancy between the annotation and the actual amino acid (AA) sequences is shown in red, the 7 AAs missing in Hop1-sv2 are highlighted in grey in the Hop1-sv1 sequence (b).

**
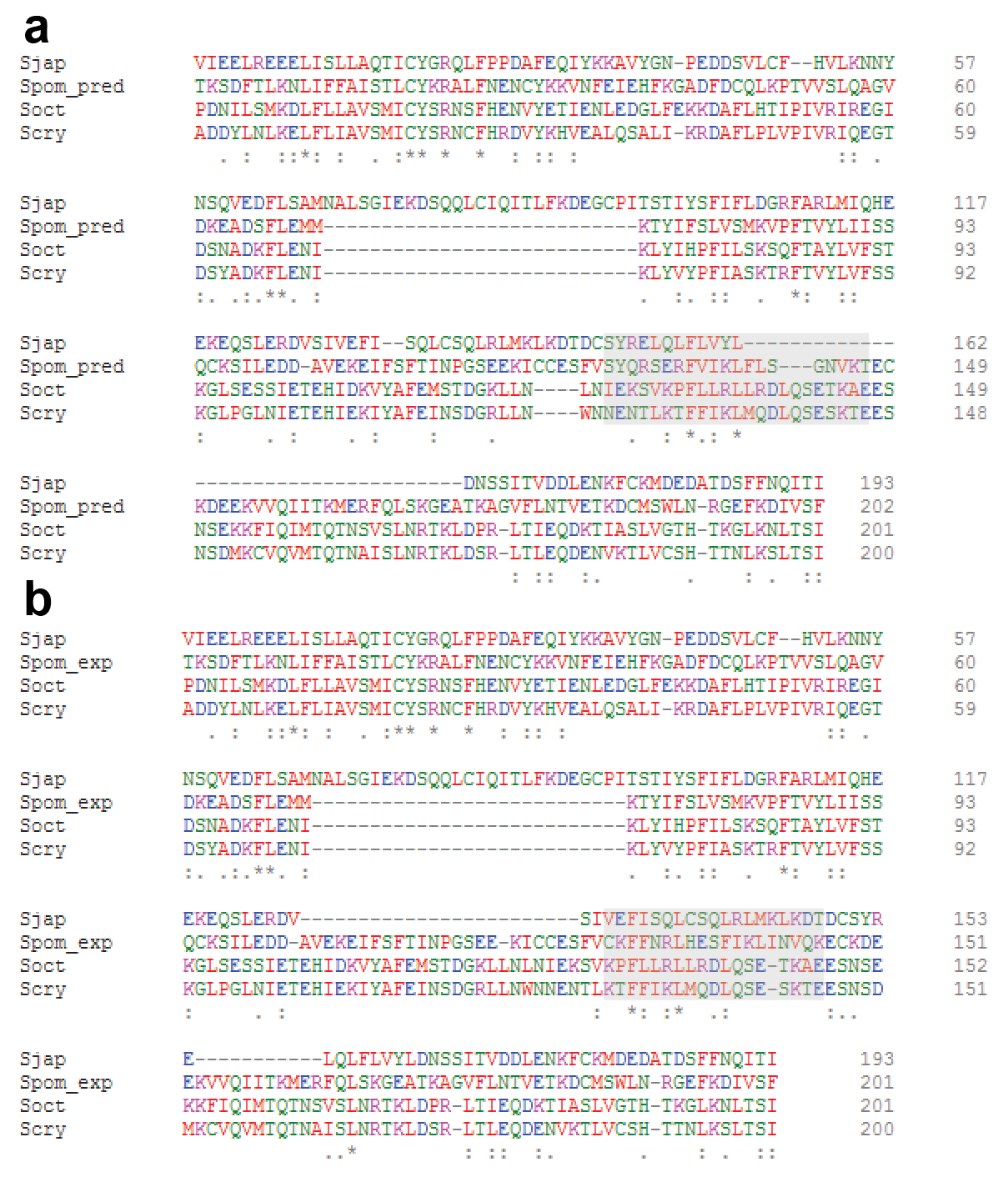
**

**Figure S3. ClustalΩ alignment** (Sievers et al. 2014) **of HORMA domains of Hop1 homologs among *Schizosaccharomyces* species.** Comparison of *Sz. japonicus* (Sjap), *Sz. octosporus* (Soct), and *Sz. cryophilus* (Scry) Hop1 homologs (HORMA domain only) against (**a**) *Sz. pombe* Hop1 HORMA domain (Spom_pred) as predicted and annotated on PomBase (<https://www.pombase.org/>, last accessed on 03/06/2017), and (**b**) *Sz. pombe* Hop1 HORMA domain (Spom_exp) as derived from experimental cDNA sequencing data of the long splice variant *hop1-sv1*. Colouring of AAs follows the standard ClustalΩ scheme according to physicochemical properties: red for small and small & hydrophobic AAs (AVFPMILW); blue for acidic AAs (DE); magenta for basic AAs (RK); and green for AAs containing hydroxyl-, sulfhydryl-, or amine-groups. Consensus symbols below the alignment indicate full conservation (*) and conservation of groups with strongly similar (:) or weakly similar (.) properties, for details see <http://www.ebi.ac.uk/Tools/msa/clustalo/help/faq.html>. The region differing between the annotation and the actual amino acid (AA) sequence for *Sz. pombe* is highlighted in grey. Numbering arbitrarily starts at the beginning of the HORMA domain and not at the beginning of the protein.


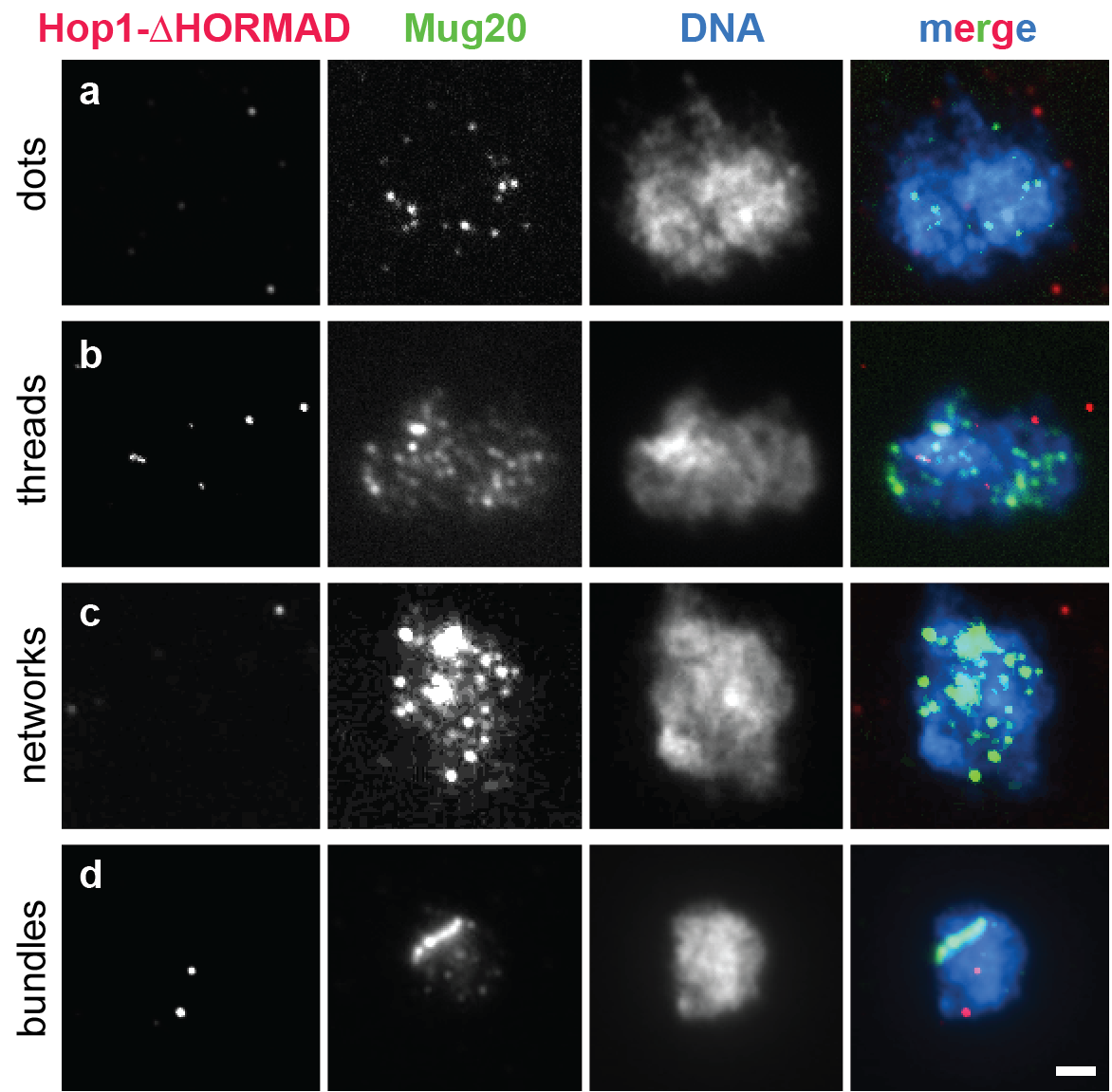


**Figure S4. Linear element morphology delineated by immunostaining of Mug20-GFP on chromatin spreads from meiotic fission yeast cells.** Linear elements as seen by immunostaining against Mug20-GFP develop from dots via threads to networks and bundles (**a**-**d**) in *h^+N^*/*h^-smt0^ hop1-*Δ*HORMAD*/*hop1-*Δ*HORMAD*::*13myc-kanMX6 mug20*^+^/*mug20*^+^::GFP*-kanMX6 ade6-M210*/*ade6-M216 ura4*^+^*/ura4-D18* (UoA947). Immunostaining against Hop1-ΔHORMAD-13myc does not localize to Mug20-GFP-positive linear elements. Mug20-GFP is shown in green, Hop1-ΔHORMAD-13myc is shown in red and Hoechst 33342-stained DNA in blue in the merge panels. Scale bar represents 2 μm.


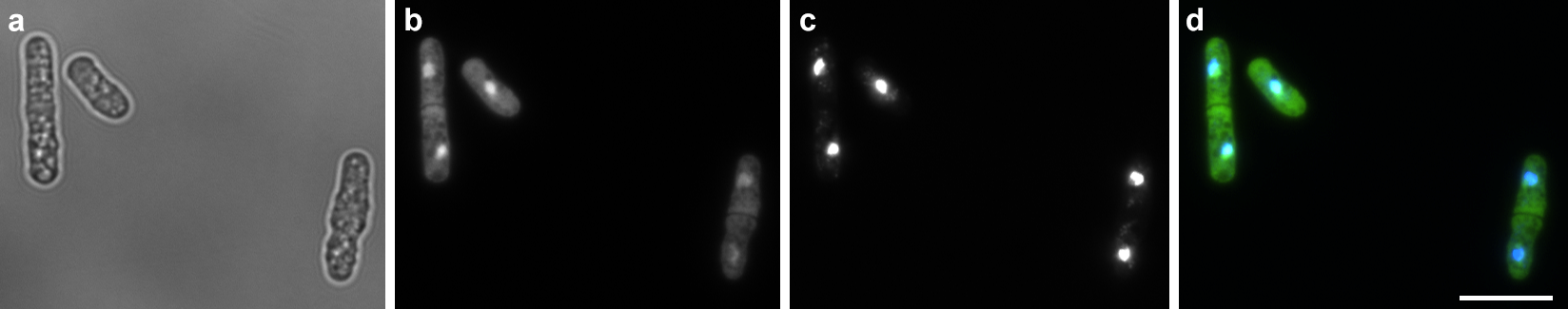


**Figure S5. Aho1-GFP localizes throughout the cytoplasm with moderate accumulation in the nucleus.** (**a**) brightfield, (**b**) GFP (green fluorescence), and (**c**) DNA stained with Hoechst 33342 (blue fluorescence) in wild-type fission yeast cells (strain FO652) over-expressing Aho1-GFP from an *nmt1*-promoter on a pREP41-type expression vector grown for 24 hours in the absence of thiamine. (**d**) overlay of (b) and (c) with GFP in green and DNA in blue. Scale bar represents 10μm.

**Table S1.** Yeast strain list (in order of appearance)

| **Strain** | **Relevant genotype** | **Origin** |
| --- | --- | --- |
| UoA399 | *h^+S^/h^-smt0^ atb2^+^/GFP-atb2^+^*::*natMX4 mug20^+^/mug20^+^*::*GFP-kanMX6 uch2^+^/uch2^+^*::*mCHERRY-ura4+ ade6-M210/ade6-M216 ura4-D18/ura4-D18* | lab strain |
| UoA722 | *h^+N^/h^-^ rad11^+^*::*GFP-kanMX6/rad11^+^ ade6-M210/ade6-M216* | lab strain |
| ALP733 | *h^+S^ ade6-3083 ura4^+^-aim2 his3-D1 leu1-32 ura4-D18* | (Lorenz et al. 2010) |
| ALP731 | *h^-smt0^ ade6-469 his3^+^-aim arg3-D4 his3-D1 ura4-D18* | (Lorenz et al. 2010) |
| UoA200 | *h^+S^ hop1*Δ*-25*::*natMX6 ade6-3083 ura4^+^-aim2 his3-D1 leu1-32 ura4-D18* | this study |
| UoA199 | *h^-smt0^ hop1*Δ*-25*::*natMX6 ade6-469 his3^+^-aim arg3-D4 his3-D1 ura4-D18* | this study |
| UoA766 | *h^+S^ hop1-sv1 ade6-3083 ura4^+^-aim2 his3-D1 leu1-32 ura4-D18* | this study |
| UoA767 | *h^-smt0^ hop1-sv1 ade6-469 his3^+^-aim arg3-D4 his3-D1 ura4-D18* | this study |
| UoA853 | *h^+S^ hop1-sv2 ade6-3083 ura4^+^-aim2 his3-D1 leu1-32 ura4-D18* | this study |
| UoA854 | *h^-smt0^ hop1-sv2 ade6-469 his3^+^-aim arg3-D4 his3-D1 ura4-D18* | this study |
| UoA945 | *h^+S^ hop1-*Δ*HORMAD ade6-3083 ura4^+^-aim2 his3-D1 leu1-32 ura4-D18* | this study |
| UoA944 | *h^-smt0^ hop1-*Δ*HORMAD ade6-469 his3^+^-aim arg3-D4 his3-D1 ura4-D18* | this study |
| UoA863 | *h^+N^ hop1-sv1 ade6-M210* | this study |
| UoA866 | *h^-smt0^ hop1-sv1*::*13myc-kanMX6 ade6-M216* | this study |
| UoA868 | *h^+N^ hop1-sv2 ade6-M210* | this study |
| UoA929 | *h^-smt0^ hop1-sv2*::*13myc-kanMX6 ade6-M216* | this study |
| UoA943 | *h^-smt0^ hop1-*Δ*HORMAD*::*13myc-kanMX6 ade6-M216* | this study |
| UoA946 | *h^+N^ hop1-*Δ*HORMAD mug20*^+^::GFP*-kanMX6 ade6-M210 ura4-D18* | this study |
| UoA878 | *h^+N^*/*h^-smt0^ hop1-sv1*/*hop1-sv1*::*13myc-kanMX6 ade6-M210*/*ade6-M216* | this study; (UoA863xUoA866) |
| UoA930 | *h^+N^*/*h^-smt0^ hop1-sv2*/*hop1-sv2*::*13myc-kanMX6 ade6-M210*/*ade6-M216* | this study; (UoA868xUoA929) |
| UoA947 | *h^+N^*/*h^-smt0^ hop1-*Δ*HORMAD*/*hop1-*Δ*HORMAD*::*13myc-kanMX6 mug20*^+^/*mug20*^+^::GFP*-kanMX6 ade6-M210*/*ade6-M216 ura4*^+^*/ura4-D18* | this study; (UoA943xUoA946) |
| FO652 | *h^-smt0^ arg3-D4 his3-D1 leu1-32 ura4-D18* | lab strain; (Lorenz et al. 2014) |
| UoA423 | *h^-smt0^ aho1*Δ*-84*::*natMX4 arg3-D4 his3-D1 leu1-32 ura4-D18* | this study |
| UoA424 | *h^+S^ aho1*Δ*-84*::*natMX4 ade6-3083 ura4^+^-aim2 his3-D1 leu1-32 ura4-D18* | this study |
| UoA427 | *h^-smt0^ aho1*Δ*-84*::*natMX4 ade6-469 his3^+^-aim arg3-D4 his3-D1 ura4-D18* | this study |
| UoA786 | *h^+S^ aho1*Δ*-84*::*natMX4 hop1*Δ*-1*::*kanMX6 ade6-3083 ura4^+^-aim2 his3-D1 leu1-32 ura4-D18* | this study |
| UoA785 | *h^-smt0^ aho1*Δ*-84*::*natMX4 hop1*Δ*-1*::*kanMX6 ade6-469 his3^+^-aim arg3-D4 his3-D1 ura4-D18* | this study |
| UoA841 | *h^-smt0^ ade6-469 his3^+^-aim his3-D1 leu1-32 ura4-D18* | this study |
| ALP812 | *h^+S^ mus81*Δ::*kanMX6* | (Lorenz et al. 2012) |
| ALP813 | *h^-smt0^ mus81*Δ::*kanMX6* | (Lorenz et al. 2012) |
| ALP802 | *h^+S^ mus81*Δ::*kanMX6 ade6-3083 ura4^+^-aim2 his3-D1 leu1-32 ura4-D18* | (Lorenz et al. 2010) |
| ALP822 | *h^-smt0^ mus81*Δ::*kanMX6 ade6-469 his3^+^-aim arg3-D4 his3-D1 ura4-D18* | (Lorenz et al. 2010) |
| UoA204 | *h^+S^ hop1*Δ*-25*::*natMX6 mus81*Δ::*kanMX6 ade6-3083 ura4^+^-aim2 his3-D1 leu1-32 ura4-D18* | this study |
| UoA203 | *h^-smt0^ hop1*Δ*-25*::*natMX6 mus81*Δ::*kanMX6 ade6-469 his3^+^-aim arg3-D4 his3-D1 ura4-D18* | this study |
| ALP1133 | *h^+S^ fml1*Δ::*hphMX4 ade6-3083 ura4^+^-aim2 his3-D1 leu1-32 ura4-D18* | (Lorenz et al. 2012) |
| FO2608 | *h^-smt0^ fml1*Δ::*hphMX4 ade6-469 his3^+^-aim arg3-D4 his3-D1 ura4-D18* | (Lorenz et al. 2012) |
| UoA182 | *h^+S^ fml1*Δ::*hphMX4 hop1*Δ*-25*::*natMX6 ade6-3083 ura4^+^-aim2 his3-D1 leu1-32 ura4-D18* | this study |
| UoA181 | *h^-smt0^ fml1*Δ::*hphMX4 hop1*Δ*-25*::*natMX6 ade6-469 his3^+^-aim arg3-D4 his3-D1 ura4-D18* | this study |
| ALP1649^a^ | *h^+S^ rad55*Δ::*hphMX ura4+-aim2 ade6-3083 his3-D1 leu1-32 ura4-D18* | (Lorenz et al. 2014) |
| ALP1648^a^ | *h^-smt0^ rad55*Δ::*hphMX his3+-aim ade6-469 arg3-D4 his3-D1 ura4-D18* | (Lorenz et al. 2014) |
| UoA847^a^ | *h^+S^ hop1*Δ*-25*::*natMX6 rad55*Δ::*hphMX4 ade6-469 his3^+^-aim arg3-D4 his3-D1 ura4-D18* | this study |
| UoA846^a^ | *h^-smt0^ hop1*Δ*-25*::*natMX6 rad55*Δ::*hphMX4 ade6-3083 ura4^+^-aim2 his3-D1 leu1-32 ura4-D18* | this study |
| ALP1623 | *h^+S^ rlp1*Δ*-7*::*natMX6 ura4+-aim2 ade6-3083 his3-D1 leu1-32 ura4-D18* | (Lorenz et al. 2014) |
| ALP1620 | *h^-smt0^ rlp1*Δ*-7*::*natMX6 his3+-aim ade6-469 arg3-D4 his3-D1 ura4-D18* | (Lorenz et al. 2014) |
| UoA849 | *h^+N^ hop1*Δ*-25*::*natMX6 rlp1*Δ::*kanMX6 ade6-469 his3^+^-aim arg3-D4 his3-D1 ura4-D18* | this study |
| UoA848 | *h^-smt0^ hop1*Δ*-25*::*natMX6 rlp1*Δ::*kanMX6 ade6-3083 ura4^+^-aim2 his3-D1 leu1-32 ura4-D18* | this study |
| ALP800 | *h^+S^ sfr1*Δ*-2*::*natMX4 ade6-3083 ura4^+^-aim2 his3-D1 leu1-32 ura4-D18* | (Lorenz et al. 2012) |
| ALP782 | *h^-smt0^ sfr1*Δ*-2*::*natMX4 ade6-469 his3^+^-aim arg3-D4 his3-D1 ura4-D18* | (Lorenz et al. 2012) |
| UoA845 | *h^+S^ hop1*Δ*-25*::*natMX6 sfr1*Δ*-11*::*hphMX4 ade6-469 his3^+^-aim arg3-D4 his3-D1 ura4-D18* | this study |
| UoA844 | *h^-smt0^ hop1*Δ*-25*::*natMX6 sfr1*Δ*-11*::*hphMX4 ade6-3083 ura4^+^-aim2 his3-D1 leu1-32 ura4-D18* | this study |
| ALP1545 | *h^+S^ dmc1*Δ*-12*::*natMX4 ade6-3083 ura4^+^-aim2 his3-D1 leu1-32 ura4-D18* | (Lorenz et al. 2012) |
| ALP1544 | *h^-smt0^ dmc1*Δ*-12*::*natMX4 ade6-469 his3^+^-aim arg3-D4 his3-D1 ura4-D18* | (Lorenz et al. 2012) |
| UoA852 | *h^+S^ dmc1*Δ­*-25*::*kanMX6 hop1*Δ*-25*::*natMX6 ade6-3083 ura4^+^-aim2 his3-D1 leu1-32 ura4-D18* | this study |
| UoA851 | *h^-smt0^ dmc1*Δ­*-25*::*kanMX6 hop1*Δ*-25*::*natMX6 ade6-469 his3^+^-aim arg3-D4 his3-D1 ura4-D18* | this study |
| UoA473 | *h^-^ ade6-149* | lab strain |
| ALP1594 | *h^-smt0^ ade7-50* *arg3-D4 his3-D1 ura4-D18* | lab strain |
| ALP1596 | *h^-smt0^ ade7-152* *his3-D1 leu1-32 ura4-D18* | lab strain; (Lorenz et al. 2014) |
| UoA725 | *h^-smt0^ hop1-3*::*ura4^+^* *arg3-D4 his3-D1 leu1-32 ura4-D18* | this study |
| UoA746 | *h^-smt0^ hop1-sv1* *arg3-D4 his3-D1 leu1-32 ura4-D18* | this study |
| UoA747 | *h^-smt0^ hop1-sv2* *arg3-D4 his3-D1 leu1-32 ura4-D18* | this study |
| UoA938 | *h^-smt0^ hop1-*Δ*HORMAD* *arg3-D4 his3-D1 leu1-32 ura4-D18* | this study |
| UoA782 | *h^-smt0^ hop1-sv1*::*13myc-kanMX6* *arg3-D4 his3-D1 leu1-32 ura4-D18* | this study |
| UoA927 | *h^+N^ hop1-sv2*::*13myc-kanMX6* *ade6-M210* | this study |
| UoA941 | *h^-smt0^ hop1-*Δ*HORMAD*::*13myc-kanMX6* *arg3-D4 his3-D1 leu1-32 ura4-D18* | this study |

^a^strains are derivatives of FY18530 provided by the National BioResource Project (NBRP) of the MEXT, Japan.

**Supplementary References**

Bähler J, Wu JQ, Longtine MS, et al (1998) Heterologous modules for efficient and versatile PCR-based gene targeting in Schizosaccharomyces pombe. Yeast 14:943–51. doi: 10.1002/(SICI)1097-0061(199807)14:10<943::AID-YEA292>3.0.CO;2-Y

Craven RA, Griffiths DJ, Sheldrick KS, et al (1998) Vectors for the expression of tagged proteins in Schizosaccharomyces pombe. Gene 221:59–68. doi: 10.1016/S0378-1119(98)00434-X

Goldstein AL, McCusker JH (1999) Three new dominant drug resistance cassettes for gene disruption in *Saccharomyces cerevisiae*. Yeast 15:1541–53. doi: 10.1002/(SICI)1097-0061(199910)15:14<1541::AID-YEA476>3.0.CO;2-K

Grimm C, Kohli J, Murray J, Maundrell K (1988) Genetic engineering of *Schizosaccharomyces pombe*: a system for gene disruption and replacement using the *ura4* gene as a selectable marker. Mol Gen Genet 215:81–6. doi: doi.org/10.1007/BF00331307

Latypov V, Rothenberg M, Lorenz A, et al (2010) Roles of Hop1 and Mek1 in meiotic chromosome pairing and recombination partner choice in *Schizosaccharomyces pombe*. Mol Cell Biol 30:1570–81. doi: 10.1128/MCB.00919-09

Lorenz A (2015) New cassettes for single-step drug resistance and prototrophic marker switching in fission yeast. Yeast 32:703–710. doi: 10.1002/yea.3097

Lorenz A (2018) Supporting Online Material - Genetic interactions between the chromosome axis-associated protein Hop1 and homologous recombination determinants in Schizosaccharomyces pombe. doi: 10.6084/m9.figshare.5373163.v2

Lorenz A, Mehats A, Osman F, Whitby MC (2014) Rad51/Dmc1 paralogs and mediators oppose DNA helicases to limit hybrid DNA formation and promote crossovers during meiotic recombination. Nucleic Acids Res 42:13723–13735. doi: 10.1093/nar/gku1219

Lorenz A, Osman F, Sun W, et al (2012) The fission yeast FANCM ortholog directs non-crossover recombination during meiosis. Science 336:1585–8. doi: 10.1126/science.1220111

Lorenz A, West SC, Whitby MC (2010) The human Holliday junction resolvase GEN1 rescues the meiotic phenotype of a *Schizosaccharomyces pombe mus81* mutant. Nucleic Acids Res 38:1866–73. doi: 10.1093/nar/gkp1179

Moreno MB, Durán A, Ribas JC (2000) A family of multifunctional thiamine-repressible expression vectors for fission yeast. Yeast 16:861–72. doi: 10.1002/1097-0061(20000630)16:9<861::AID-YEA577>3.0.CO;2-9

Sato M, Dhut S, Toda T (2005) New drug-resistant cassettes for gene disruption and epitope tagging in *Schizosaccharomyces pombe*. Yeast 22:583–91. doi: 10.1002/yea.1233

Sievers F, Wilm A, Dineen D, et al (2014) Fast, scalable generation of high-quality protein multiple sequence alignments using Clustal Omega. Mol Syst Biol 7:539–539. doi: 10.1038/msb.2011.75
